# 42.2 INFLAMMATION AND GUT MICROBIOME IN FIRST-EPISODE PSYCHOSIS

**DOI:** 10.1093/schbul/sby014.175

**Published:** 2018-04-01

**Authors:** Jaana Suvisaari, Outi Mantere, Jaakko Keinänen, Tuula Kieseppä, Maria Saarela, Robert Yolken, Jarno Honkanen

**Affiliations:** 1 National Institute for Health and Welfare; 2 McGill University; 3 Helsinki University Hospital; 4 VTT Technical Research Centre of Finland; 5 Johns Hopkins University; 6 University of Helsinki

## Abstract

**Background:**

Patients with first-onset psychosis have evidence of impaired glucose tolerance, but otherwise are metabolically healthy when traditional cardiovascular risk markers are used. After antipsychotic treatment is started, there is rapid weight gain and emergence of dyslipidemias. Weight gain and development of abdominal obesity is accompanied by worsening chronic low-grade inflammation. Activation of innate immunity is often present at the onset of disease. One unexplored mechanism possibly contributing to these problems is altered gut microbiota.

**Methods:**

The Helsinki Early Psychosis Study recruited 97 patients with first-episode psychosis and 62 controls into a longitudinal study. Here, data on longitudinal changes in inflammation, weight gain and abdominal obesity during the first year of treatment in patients with first-episode psychosis is presented and compared with matched healthy controls. Three time points (baseline, 2 months, 12 months) are available for patients and two (baseline, 12 month) for controls. The possible contribution of different antipsychotics will be explored, and whether patients who were no longer using antipsychotics at the one-year follow-up have less problems in these measures. First results regarding the gut microbiome will be presented (Schwarz et al. 2017), and the possible contribution of gut microbiota to inflammation and weight gain in first-episode psychosis explored.

**Results:**

Our previous findings from a subset of the study sample found most marked changes in innate immunity chemokines (Mäntylä et al. 2015), whereas full longitudinal data on 38 cyto- and chemokines will be available at the SIRS congress. As a preliminary result on the longitudinal course of inflammation, high-sensitivity C-reactive protein showed a significant increase during the first year of treatment in patients (median baseline 0.65 mg/l, 2 months 0.79 mg/l and 12 months 1.68 mg/l). Data on PBMC gene expression will also be presented, revealing notable differences related to different antipsychotic use.

**Discussion:**

The findings will be discussed in the context of to what extent they may reflect underlying disease mechanisms and environmental contributions, including gut microbiota alterations, and to what extent inflammation is a secondary phenomenon related to antipsychotic use and weight gain.

**References:**

1) Mäntylä T, Mantere O, Raij TT, Kieseppä T, Laitinen H, Leiviskä J, Torniainen M, Tuominen L, Vaarala O, Suvisaari J. Altered activation of innate immunity associates with white matter volume and diffusion in first-episode psychosis. PLoS One. 2015 May 13;10(5):e0125112.

2)Schwarz E, Maukonen J, Hyytiäinen T, Kieseppä T, Orešič M, Sabunciyan S, Mantere O, Saarela M, Yolken R, Suvisaari J. Analysis of microbiota in first episode psychosis identifies preliminary associations with symptom severity and treatment response. Schizophr Res. 2017 [Epub ahead of print]

